# Knowledge, Attitudes, and Perceptions Regarding CPR Among Non-medical Staff at a Medical School in South Africa

**DOI:** 10.7759/cureus.33506

**Published:** 2023-01-08

**Authors:** Saeb Jarghon, Kamo Molokoane, Abdullah E Laher, Feroza Motara

**Affiliations:** 1 Emergency Medicine, University of the Witwatersrand, Johannesburg, ZAF

**Keywords:** cpr perceptions, cpr attitude, cpr knowledge, medical school, non-medical staff, cpr, cardiopulmonary resuscitation

## Abstract

Background

Sudden cardiac arrest can occur unexpectedly in any person and at any place including at medical schools. Improved outcomes after cardiac arrest are dependent on the initiation of early first responder high-quality cardiopulmonary resuscitation (CPR) and rapid defibrillation. There is a lack of data pertaining to the knowledge, attitudes, and perceptions of non-medical staff at medical schools regarding CPR. The aim of this study was to determine the knowledge, attitudes, and perceptions of non-medical staff employed at a medical school in South Africa regarding CPR.

Methods

In this cross-sectional survey study, a paper-based questionnaire was administered to non-medical staff (i.e., all staff without a medical [MBBCh or equivalent] or nursing degree) employed at the medical school. Data were collected between August 1 and October 25, 2020.

Results

The final study sample comprised 150 participants. Of these, 103 (68.7%) were female, 109 (72.7%) were ≤ 40 years old, 62 (41.3%) had a postgraduate university degree, 72 (48.0%) had witnessed a medical emergency at the medical school premises and 46 (30.7%) had previously undertaken first aid or CPR training. The mean (SD) knowledge score was 4.4 ± 1.6 out of 10 with only 25 (16.7%) participants knowing what the first thing was to look out for during a medical emergency and 28 (18.7%) participants knowing the location of the automated external defibrillator. Most participants (n=136, 90.7%) indicated that CPR training should be mandatory for all employees.

Conclusion

Non-medical staff surveyed displayed suboptimal knowledge but positive attitudes and perceptions toward CPR. Although this was a single-center study, these results can be used to motivate CPR training of non-medical staff at all medical schools.

## Introduction

Life-threatening emergencies including sudden cardiac arrest (SCA) can occur at any place, either in- or out-of-hospital, and accounts for the majority of deaths globally with survival rates varying significantly from region to region [1]. Annually, out-of-hospital cardiac arrest (OHCA) is responsible for approximately 350,000 deaths in Europe and another 300,000 deaths in the United States [2,3]. The global survival rate of OHCA remains dismal at 8.8% [4]. Mortality for the most part is determined by the time lag between the onset of cardiac arrest and initiation of high-quality cardiopulmonary resuscitation (CPR) and the arrival of emergency medical services (EMS) [5].

SCA has various etiologies, with coronary artery disease representing up to 80% of cases [6]. Studies among various segments of the South African population have revealed a high burden of risk factors for cardiovascular disease, which is likely to be associated with an increased risk of SCA [7]. The annual prevalence of OHCA in South Africa is estimated at 23.2 per 100,000 persons [8].

The American Heart Association’s (AHA) chain of survival for OHCA comprises six links: namely 1) recognition of cardiac arrest and activation of the emergency response system, 2) early CPR, 3) rapid defibrillation, 4) advanced resuscitation by EMS, 5) post-cardiac arrest care and 6) recovery [9]. The first three links can be initiated by any first responder on the scene who may be a trained medical person or a bystander. Minimum bystander CPR requirements include the ability to recognize a person in cardiac arrest, call for help, and initiate timeous hands-only chest compressions [10]. Barriers to laypersons performing CPR include a lack of training and fears of causing more harm than good [11].

Medical schools are staffed by medical and support non-medical personnel such as lecturers, administration staff, security staff, cleaners, librarians, etc., who can be regarded as laypersons. Knowing that cardiovascular disease is a common cause of death in South Africa and its strong association with SCA, it is imperative that laypersons are capable of performing the first three links of the AHA chain of survival. Previous studies have reported on the knowledge, attitudes, and perceptions of medical students and healthcare professionals regarding CPR, however, there is a lack of studies pertaining to this among non-medical staff at medical schools [12,13]. Hence, we aimed to determine the knowledge, attitudes, and perceptions regarding CPR of non-medical staff at a medical school in South Africa.

## Materials and methods

This was a cross-sectional questionnaire-based study. The study population comprised non-medical staff who were employed at the medical school (Faculty of Health Sciences) premises of an established university situated in South Africa. The Faculty of Health Sciences has seven schools (divisions) namely the School of Anatomical Sciences, School of Clinical Medicine, School of Oral Health Sciences, School of Pathology, School of Physiology, School of Public Health, and School of Therapeutic Sciences. At the time that the study was conducted, a total of 269 non-medical staff were employed at the time that the study was conducted. Permission to conduct the study was granted by the Dean of the Faculty of Health Sciences. Ethics approval was obtained from the Human Research Ethics Committee of the University of Witwatersrand (certificate clearance no. M200632).

Study participation was voluntary. We aimed to enroll a convenience sample of at least 150 staff members. Excepting staff with a medical degree (MBBCh or equivalent) or nursing degree, all other staff members were eligible to participate. The questionnaire (appendices - Tables 2, 3) was developed by the study investigators and included a total of 22 questions that pertained to sociodemographic characteristics (five questions), medical emergencies previously encountered at the medical school premises (one question), previous first aid or CPR training (one question), barriers to CPR training (one question), knowledge pertaining to CPR (10 questions) and attitudes and perceptions toward the practice of CPR (four questions). A knowledge score was determined for each participant. This was calculated by allocating one point for each of the 10 knowledge questions that were correctly answered. The mean (SD) knowledge score for the entire cohort was thereafter determined.

Data were collected between August 1 and October 25, 2020. Potential study participants were first handed a study information leaflet that summarized the study aims and objectives. After confirming eligibility for study participation and obtaining informed consent, the paper-based study questionnaire was handed over to the participant for completion. To preserve participant confidentiality, no identifying information was collected.

Collected data were thereafter captured into an electronic data spreadsheet (Microsoft 365, Version 16.0.13029.20232) and analyzed. Since most of the data were descriptive in nature, these were presented using frequency and percentage tables and graphs. To determine the CPR knowledge score, one point was given for each of the 10 questions that were answered correctly. We thereafter calculated the mean and standard deviation of the score for the entire cohort of participants.

## Results

Of the 158 questionnaires that were distributed, a total of 150 (94.9%) were completed. Table 1 describes the characteristics of the study participants. In total, 103 (68.7%) were female, 109 (72.7%) were ≤ 40 years old, 62 (41.3%) had a postgraduate university degree, 86 (57.6%) were lecturers, and 88 (58.7%) were employed at the university for <5 years. A total of 72 (48.0%) participants reported that they had witnessed a medical emergency at the medical school premises. Among the medical emergencies witnessed, difficulty breathing, collapse, and seizures accounted for the most common conditions that were witnessed by 47 (31.3%), 44 (29.3%), and 30 (20.0%) participants, respectively. Overall, 46 (30.7%) participants had previously undertaken first aid or CPR training.

**Table 1:** Characteristics of study participants CPR – cardiopulmonary resuscitation

	n (%)
Sex	
Female	103 (68.7)
Male	47 (31.3)
Age category	
≤ 40 years	109 (72.7)
> 40 years	41 (27.2)
Highest level of education	
Did not complete matric	2 (1.3)
Matric certificate	16 (10.7)
Diploma	23 (15.3)
Undergraduate degree	47 (31.3)
Postgraduate degree	62 (41.3)
Position of employment	
Lecturer	86 (57.3)
Administrator	43 (28.7)
Other (security, cleaners, library, etc.)	21 (14.0)
Duration of current employment	
< 6 months	7 (4.7)
6-12 months	20 (13.3)
1-5 years	61 (40.7)
5-10 years	20 (13.3)
>10 years	42 (28.0)
Medical emergencies witnessed on medical school premises	
Difficulty breathing (e.g., asthma attack)	47 (31.3)
Collapse	44 (29.3)
Seizures (fits, convulsions)	30 (20.0)
Choking	20 (13.3)
Heart attack	10 (6.7)
Stroke	7 (4.7)
Previously undertook first-aid or CPR training	46 (30.7)

**Figure 1:**
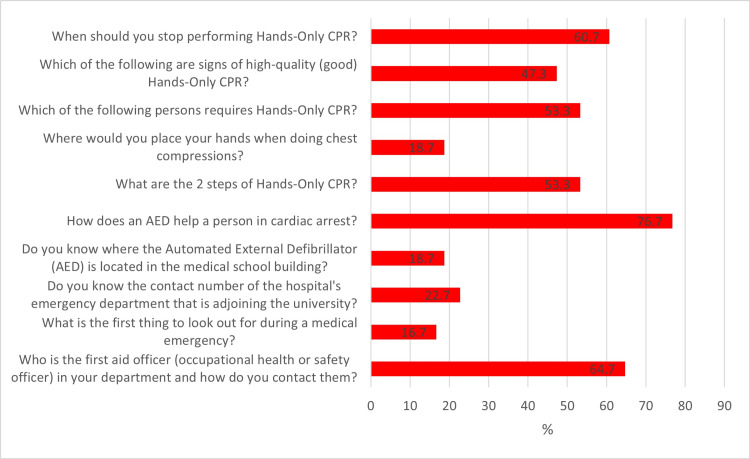
Percentage of correct answers for each of the CPR knowledge questions CPR – cardiopulmonary resuscitation

Figure 1 describes the percentage of correct answers for each of the CPR knowledge questions. Of note, only 25 (16.7%) participants knew what was the first thing to look out for during a medical emergency, only 28 (18.7%) knew where the automated external defibrillator (AED) was located in the medical school building, only 28 (18.7%) knew where to place their hands when doing chest compressions and only 34 (22.7%) knew the contact number of the hospital emergency department adjoining the medical school. Of the 10 CPR knowledge questions, the mean (SD) a number of correct answers for the entire cohort was 4.4 ± 1.6.

**Figure 2:**
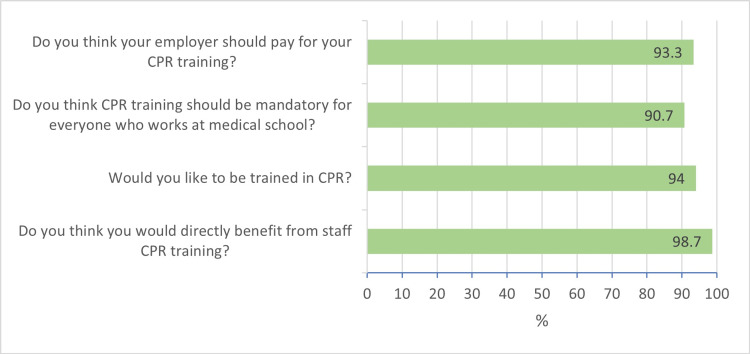
Attitudes and perceptions of study participants toward the practice of CPR CPR – cardiopulmonary resuscitation

Figure 2 depicts the attitudes and perceptions of participants toward the practice of CPR. Almost all participants thought that they would directly benefit from staff CPR training (n=148, 98.7%), would like to be trained in CPR (n=141, 94.0%), thought that CPR training should be mandatory for everyone working at medical school (n=136, 90.7%) and thought that their employer should pay for their CPR training course (n=140, 93.3%).

**Figure 3:**
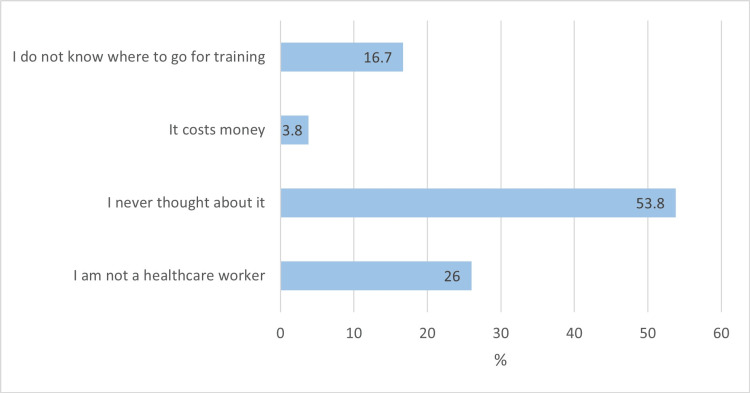
Barriers to CPR training among the 104 CPR untrained participants

Barriers to CPR training are described in Figure 3. Among the 104 (69.3%) participants who were not CPR trained, the two most common reasons were that they never thought about it (n=56, 53,8%) and that they were not a healthcare worker (n=27, 26.0%).

## Discussion

To our knowledge, this is the first study to have evaluated the knowledge, attitudes, and perceptions of non-medical staff at a medical school in South Africa regarding CPR. In this study, 48% of participants reported that they had witnessed a medical emergency at the medical school premises, with breathing difficulty, collapse, convulsions, choking, heart attack, and stroke being reported among the conditions that were witnessed. Comparatively, in a similar design study by Ojifinni et al., among student teachers in South Africa, participants reported that trauma-related conditions, allergic reactions, breathing difficulties, convulsions, loss of consciousness, choking, and drowning were among the medical conditions that were witnessed during school-based practice teaching sessions [14]. According to the Global Initiative for Asthma (GINA), South Africa has the fourth-highest asthma-related mortality in the world [15], while there are more than 200 reported cardiovascular and stroke-related deaths in South Africa every day [16]. Hence, it is recommended that CPR training courses also include basic educational material pertaining to commonly witnessed medical emergencies and the steps to be taken while awaiting expert medical assistance.

In this study, approximately a third (30.9%) of participants reported participating in a first aid or CPR training course. The overall mean CPR knowledge score was 4.4 ± 1.6 out of the 10 survey questions in this category, with participants scoring poorly in questions pertaining to recognizing a medical emergency, location of the AED, and chest compression method. Comparatively, a study conducted by Hung et al., among college students in Hong Kong reported that 55.3% of participants had previous CPR training and the mean CPR knowledge score was 4.97 (SD 1.61) out of 10, with participants scoring poorly in questions pertaining to CPR sequence, bystander action, and chest compression method [17]. In the study by Ojifinni et al., the mean knowledge score was 4.0 ± 1.7 out of 12 with questions pertaining to recognizing a medical emergency, the correct steps of using an AED, and the method of CPR scoring low [14]. Another study conducted by Alotaibi et al., among dental students and staff at a university in Saudi Arabia, reported that 99.1% of participants had previously undertaken CPR training, and the mean knowledge score was 5.99 out of 12 with participants scoring poorly in questions pertaining to CPR in new-born infants and the management of a choking adult [18]. Hence, our findings are in keeping with general international trends, which indicates that CPR knowledge among individuals at tertiary education institutes is generally poor. This calls for all tertiary institutes to implement measures and policies that will improve CPR knowledge and skill. These measures should include training of a higher number of staff/students and ensuring regular re-training.

In this study, almost all participants indicated that they would benefit from and would like to be trained in CPR, that CPR training should be mandatory for all medical school employees and that the employer should be responsible for covering the cost of CPR training. Although it is desirable that CPR training is offered to all individuals at the workplace, the Occupational Health and Safety Act of South Africa stipulates that where there are more than 10 employees, there should be a trained person at the workplace premises who is able to assist with a medical emergency, with shops and offices requiring at least one trained person per 100 employees, and other entities requiring at least one trained person per 50 employees. While it would be regarded as a due courtesy for the employer to cover the full cost or at least partially subsidize the cost of employee CPR training, the employer is by no means bound to cover such costs and may stipulate that CPR or first aid certification be a condition of employment [19,20]. We however recommend that employers should explore other options such as video-based CPR self-instruction, which is timesaving, less expensive, can be repeated more frequently, and may be more effective than traditional classroom-based CPR training courses [21,22].

In this study, barriers to undertaking CPR training among the 69.3% of CPR untrained participants were reported as not having thought about it (53.8%), not being a healthcare worker (26.0%), not knowing where to train (16.7%) and cost (3.8%). Comparatively, a study among 883 allied health university students in Jordan, reported that 81.8% of participants were untrained, with not knowing where to train (33.0%), lack of time (32.1%), cost (14.1%), lack of interest (12.3%) and lack of availability (8.4%) being reported as barriers to CPR training [23]. A qualitative study that enrolled 25 school leadership members and teachers from eight schools in Denmark identified three key themes as barriers to CPR training. These were described as insecurity about one's own CPR instruction skills, insecurity about one's own CPR skills, and organization of CPR training [24]. Another study that was conducted among six neighborhoods with relatively high rates of OHCA in Ohio, USA, reported that financial cost, lack of information, and fear of self-harm were the major barriers to CPR training [25]. Yet another mixed qualitative and quantitative survey study comprising 137 laypersons in the USA reported that fear of performing CPR incorrectly, the concern of causing injury to the patient, and fear of being sued were the most common barriers to CPR training [26]. In the study by Ojifinni et al., not knowing where to train (76.4%), not being a healthcare worker (59.0%), not thinking about it (36.9%) and cost (29.9%) were reported as major barriers to CPR training [14]. Hence, there is a need to initiate public awareness programs that will focus on allaying fears and concerns regarding CPR training.

There are some limitations to this study. Firstly, the study was conducted at a single tertiary institute, hence, our results cannot be generalized to other institutes in South Africa. Secondly, since the study was conducted during the peak of the COVID-19 pandemic with there being modified staff working schedules in place, the number of security staff, cleaning staff, library staff, etc., who were available to participate in the study was low, which may also have influenced our study findings. Other potential limitations of this study include recall bias and also the lack of validation of our survey questions. Nevertheless, we are hopeful that the findings of our study will encourage similar studies at other tertiary educational institutes and also serve to enhance the knowledge, attitudes, and perceptions regarding CPR in general.

## Conclusions

Our study findings are in keeping with general international trends. A substantial proportion of the study participants had witnessed a medical emergency at the study site. Participants however displayed suboptimal knowledge but positive attitudes and perceptions toward CPR. Although this was a single-center study, these results can be used to motivate CPR training of non-medical staff at all tertiary educational institutes. Future studies should also aim to determine the knowledge, attitudes, and perceptions regarding CPR at other institutes locally.

## Appendices

Study questionnaire

**Table 2:** Section A of the study questionnaire Please indicate the appropriate responses with X CPR – cardiopulmonary resuscitation

	Response
What is your sex	
Male	
Female	
What is your age?	
≤ 40 years	
> 40 years	
What is your highest level of education?	
Did not complete matric	
Matric certificate	
Diploma	
Undergraduate degree	
Postgraduate degree	
What is your current position of employment?	
Lecturer	
Administrator	
Other (security, cleaners, library, etc.)	
What is your duration of employment at the medical school?	
< 6 months	
6-12 months	
1-5 years	
5-10 years	
>10 years	
Have you ever witnessed any of the medical emergencies listed below during the course of your employment at the medical school (You may circle more than one option where appropriate)?	
Someone suffering from a stroke	
Someone suffering from a heart attack	
Someone choking (a blockage of the upper breathing pipe by food or other objects)	
Someone having difficulty breathing	
Someone having seizures (fits, convulsions)	
Someone collapsing	
Have you previously been trained in first-aid or CPR?	
Yes	
No	
If you have not previously been trained in CPR, which of the following would you regard as barriers to CPR training (You may circle more than one option where appropriate)?	
I do not know where to go for the training	
It costs money	
I never thought about it	
I am not a health care provider	

**Table 3:** Section B of the study questionnaire Please indicate the appropriate responses with X CPR – cardiopulmonary resuscitation

	Response
When should you stop performing Hands-Only CPR?	
When emergency medical staff arrive	
If the person speaks, moves, or breathes normally	
If the scenario becomes dangerous	
All of the above	
Which of the following are signs of high-quality (good) Hands-Only CPR?	
Push Hard	
Push Fast	
Push at a rate of 100-120 compressions per minute	
All of the above	
Which of the following persons requires Hands-only CPR?	
A person with a pulse but trouble breathing	
A person who is unresponsive with no normal breathing and no pulse	
A person who is unresponsive but is breathing normally	
I don’t know	
Where would you place your hands when doing chest compressions?	
Upper part of the breastbone	
Middle of the breastbone	
Lower part of the breastbone	
I don’t know	
What are the 2 steps of Hands-only CPR?	
Call for help and start chest compressions	
Call for help and wait for the emergency medical services to arrive	
Provide chest compressions and mouth-to-mouth breaths	
I don’t know	
How does an Automated External Defibrillator (AED) help a person in cardiac arrest?	
An AED pumps blood	
An AED shocks the brain	
An AED helps the victim breathe	
An AED restores normal heart rhythm	
Do you know where the Automated External Defibrillator (AED) is located in the medical school building?	
Yes	
No	
Do you know the contact number for the hospital’s emergency department that is adjoining the university?	
Yes	
No	
What is the first thing to look out for during a medical emergency?	
If the person is unconscious	
If the person is breathing and has a pulse	
If I am in danger	
I don’t know	
Do you know who the first aid officer (occupational health and safety officer) in your department is and how to contact them?	
Yes	
No	
Do you think your employer should pay for your CPR training?	
Yes	
No	
Do you think CPR training should be mandatory for everyone who works at medical school?	
Yes	
No	
Would you like to be trained in CPR?	
Yes	
No	
Do you think you would directly benefit from staff CPR training?	
Yes	
No	

## References

[1] Wong CX, Brown A, Lau DH, Chugh SS, Albert CM, Kalman JM, Sanders P (2019). Epidemiology of sudden cardiac death: global and regional perspectives. Heart Lung Circ.

[2] Berdowski J, Berg RA, Tijssen JG, Koster RW (2010). Global incidences of out-of-hospital cardiac arrest and survival rates: systematic review of 67 prospective studies. Resuscitation.

[3] Roger VL, Go AS, Lloyd-Jones DM (2011). Heart disease and stroke statistics--2011 update: a report from the American Heart Association. Circulation.

[4] Yan S, Gan Y, Jiang N (2020). The global survival rate among adult out-of-hospital cardiac arrest patients who received cardiopulmonary resuscitation: a systematic review and meta-analysis. Crit Care.

[5] Mills EH, Aasbjerg K, Hansen SM (2019). Prehospital time and mortality in patients requiring a highest priority emergency medical response: a Danish registry-based cohort study. BMJ Open.

[6] Chin A (2014). Sudden cardiac death in Africa. Cardiovasc J Afr.

[7] Schutte AE (2019). Urgency for South Africa to prioritise cardiovascular disease management. Lancet Glob Heal.

[8] Stassen W, Wylie C, Djärv T, Wallis LA (2021). Out-of-hospital cardiac arrests in the city of Cape Town, South Africa: a retrospective, descriptive analysis of prehospital patient records. BMJ Open.

[9] Berg KM, Cheng A, Panchal AR (2020). Part 7: Systems of care: 2020 American Heart Association guidelines for Cardiopulmonary Resuscitation and Emergency Cardiovascular Care. Circulation.

[10] Sayre MR, Berg RA, Cave DM, Page RL, Potts J, White RD (2008). Hands-only (compression-only) cardiopulmonary resuscitation: a call to action for bystander response to adults who experience out-of-hospital sudden cardiac arrest: a science advisory for the public from the American Heart Association Emergency Cardiovascular Care Committee. Circulation.

[11] Dobbie F, Angus K, Uny I, Duncan E, MacInnes L, Hasseld L, Clegg G (2018). Protocol for a systematic review to identify the barriers and facilitators to deliver bystander cardiopulmonary resuscitation (CPR) in disadvantaged communities. Syst Rev.

[12] Mohammed Z, Arafa A, Saleh Y (2020). Knowledge of and attitudes towards cardiopulmonary resuscitation among junior doctors and medical students in Upper Egypt: cross-sectional study. Int J Emerg Med.

[13] Rajeswaran L, Cox M, Moeng S, Tsima BM (2018). Assessment of nurses' cardiopulmonary resuscitation knowledge and skills within three district hospitals in Botswana. Afr J Prim Health Care Fam Med.

[14] Ojifinni K, Motara F, Laher AE (2019). Knowledge, attitudes and perceptions regarding basic life support among teachers in training. Cureus.

[15] Masoli M, Fabian D, Holt S, Beasley R (2004). The global burden of asthma: executive summary of the GINA Dissemination Committee report. Allergy.

[16] (2022). Mortality and causes of death in South Africa: Findings from death notification. https://www.google.com/url?sa=t&rct=j&q=&esrc=s&source=web&cd=&ved=2ahUKEwiBrM-ox8v2AhWu8bsIHQopBwUQFnoECAMQAQ&url=https%3A%2F%2Fwww.statssa.gov.za%2Fpublications%2FP03093%2FP030932018.pdf&usg=AOvVaw2iOO-JSn9qz7bROZo8q8cM.

[17] Hung MSY, Chow MCM, Chu TTW (2017). College students’ knowledge and attitudes toward bystander cardiopulmonary resuscitation: a cross-sectional survey. Cogent Med.

[18] Alotaibi O, Alamri F, Almufleh L, Alsougi W (2016). Basic life support: knowledge and attitude among dental students and staff in the college of dentistry, King Saud University. Saudi J Dent Res.

[19] First-Aid Training SA (2022). First-Aid Training SA. https://www.firstaidtrainingsa.co.za/faq/.

[20] The Future of First Aid Training (Update 8 (2022). The Future of First Aid Training (Update 8). https://www.saiosh.co.za/news/556944/The-Future-of-First-Aid-Training-Update-8.htm.

[21] Lynch B, Einspruch EL, Nichol G, Becker LB, Aufderheide TP, Idris A (2005). Effectiveness of a 30-min CPR self-instruction program for lay responders: a controlled randomized study. Resuscitation.

[22] Del Rios M, Han J, Cano A, Ramirez V, Morales G, Campbell TL, Hoek TV (2018). Pay it forward: High School video-based instruction can disseminate CPR knowledge in priority neighborhoods. West J Emerg Med.

[23] Oteir AO, Almhdawi KA, Kanaan SF, Alwidyan MT, Williams B (2019). Cardiopulmonary resuscitation level of knowledge among allied health university students in Jordan: a cross-sectional study. BMJ Open.

[24] Zinckernagel L, Malta Hansen C, Rod MH, Folke F, Torp-Pedersen C, Tjørnhøj-Thomsen T (2016). What are the barriers to implementation of cardiopulmonary resuscitation training in secondary schools? A qualitative study. BMJ Open.

[25] Sasson C, Haukoos JS, Bond C (2013). Barriers and facilitators to learning and performing cardiopulmonary resuscitation in neighborhoods with low bystander cardiopulmonary resuscitation prevalence and high rates of cardiac arrest in Columbus, OH. Circ Cardiovasc Qual Outcomes.

[26] Blewer AL, Leary M, Fredericks AC (2010). Self-reported barriers to CPR education among laypersons offered training. Circulation.

